# The Omicron Lineages BA.1 and BA.2 (*Betacoronavirus* SARS-CoV-2) Have Repeatedly Entered Brazil through a Single Dispersal Hub

**DOI:** 10.3390/v15040888

**Published:** 2023-03-30

**Authors:** Alessandra P. Lamarca, Ueric José Borges de Souza, Filipe Romero Rebello Moreira, Luiz G. P. de Almeida, Mariane Talon de Menezes, Adrieli Barboza de Souza, Alessandro Clayton de Souza Ferreira, Alexandra L. Gerber, Aline B. de Lima, Ana Paula de C. Guimarães, Andréa Cony Cavalcanti, Aryel B. Paz e Silva, Bruna Israel Lima, Cirley Lobato, Cristiane Gomes Da Silva, Cristiane P. T. B. Mendonça, Daniel Costa Queiroz, Danielle Alves Gomes Zauli, Diego Menezes, Fábio Sossai Possebon, Franciano Dias Pereira Cardoso, Frederico Scott Varella Malta, Isabela Braga-Paz, Joice do Prado Silva, Jorge Gomes Goulart Ferreira, Jucimária Dantas Galvão, Leandro Magalhães de Souza, Leonardo Ferreira, Lia Gonçalves Possuelo, Liliane Tavares de Faria Cavalcante, Luige B. Alvim, Luiz Fellype Alves de Souza, Luiza C. G. de Araújo E Santos, Rillery Calixto Dias, Rutilene Barbosa Souza, Thaís Regina y Castro, Andréia Rosane de Moura Valim, Fabrício Souza Campos, João Pessoa Araujo, Priscila de Arruda Trindade, Renato S. Aguiar, Robson Michael Delai, Ana Tereza R de Vasconcelos

**Affiliations:** 1 Laboratório de Bioinformática, Laboratório Nacional de Computação Científica, Petrópolis 25651-075, Brazil; 2 Laboratório de Bioinformática e Biotecnologia, Universidade Federal do Tocantins, Campus de Gurupi, Palmas 77410-570, Brazil; 3 Laboratório de Virologia Molecular, Departamento de Genética, Instituto de Biologia, Universidade Federal do Rio de Janeiro, Rio de Janeiro 21941-901, Brazil; 4 Centro de Medicina Tropical da Tríplice Fronteira, Foz do Iguaçu 85866-010, Brazil; 5 Departamento de Pesquisa & Desenvolvimento, Instituto Hermes Pardini, Belo Horizonte 30140-070, Brazil; 6 Laboratório Central de Saúde Pública Noel Nutels, Rio de Janeiro 20231-092, Brazil; 7 Departamento de Genética, Ecologia e Evolução, Instituto de Ciências Biológicas, Universidade Federal de Minas Gerais, Belo Horizonte 31270-901, Brazil; 8 Laboratório de Biologia Molecular, Parque Científico e Tecnológico Regional, Universidade de Santa Cruz do Sul, Santa Cruz do Sul 96815-900, Brazil; 9 Centro de Ciências de Saúde e do Desporto, Universidade Federal do Acre, Rio Branco 69920-900, Brazil; 10 Instituto de Biotecnologia, Universidade Estadual Paulista, Botucatu 18618-689, Brazil; 11 Laboratório Central de Saúde Pública do Estado do Tocantins, Palmas 77054-970, Brazil; 12 Departmento de Ciências da Vida, Universidade de Santa Cruz do Sul, Santa Cruz do Sul 96815-900, Brazil; 13 Centro de Infectologia Charles Mérieux and Laboratório Rodolphe Mérieux, Hospital das Clínicas do Acre, Rio Branco 69920-223, Brazil; 14 Laboratório de Biologia Molecular e Bioinformática Aplicadas a Microbiologia Clínica, Universidade Federal de Santa Maria, Santa Maria 97105-900, Brazil; 15 Laboratório de Virologia, Departamento de Microbiologia, Imunologia e Parasitologia, Instituto de Ciências Básicas da Saúde, Universidade Federal do Rio Grande do Sul, Porto Alegre 90010-150, Brazil

**Keywords:** COVID-19, phylodynamics, variant of concern, genome, viral evolution, dispersal

## Abstract

Brazil currently ranks second in absolute deaths by COVID-19, even though most of its population has completed the vaccination protocol. With the introduction of Omicron in late 2021, the number of COVID-19 cases soared once again in the country. We investigated in this work how lineages BA.1 and BA.2 entered and spread in the country by sequencing 2173 new SARS-CoV-2 genomes collected between October 2021 and April 2022 and analyzing them in addition to more than 18,000 publicly available sequences with phylodynamic methods. We registered that Omicron was present in Brazil as early as 16 November 2021 and by January 2022 was already more than 99% of samples. More importantly, we detected that Omicron has been mostly imported through the state of São Paulo, which in turn dispersed the lineages to other states and regions of Brazil. This knowledge can be used to implement more efficient non-pharmaceutical interventions against the introduction of new SARS-CoV variants focused on surveillance of airports and ground transportation.

## 1. Introduction

The zoonotic origin of the severe acute respiratory syndrome Coronavirus 2 (SARS-CoV-2) and its spillover to humans make it clear that our androgenetic approach to infectious diseases needs to be abandoned [1]. The virus caused the greatest pandemic of the 21st century so far [2], with more than 6.6 million resulting deaths as of the beginning of December 2022 [3]. Since its emergence and spread around the world, the “Coronavirus disease 2019” (COVID-19) has had different socio-economic impacts on the worldwide population, depending on the way in which each country has dealt with the pandemic [4,5,6]. The efforts needed to combat SARS-CoV-2 were insufficient in many countries and resulted in a high number of cases and deaths. Brazil has suffered more than 689,000 casualties due to COVID-19, ranking second in absolute deaths as of 1 December 2022. Even considering the population size, Brazil had the 19th highest number of deaths per million on the same date.

One of the major hindrances to controlling the pandemic in Brazil is the continental proportions of the country. The unequal population distribution in this gigantic territory creates challenges ranging from testing and vaccinating in remote areas to creating a coordinated response to the pandemic across the country [7,8,9,10]. Reaching the most distant areas to vaccinate the Brazilian people was efficiently tackled by the largest government-run public health care system in the world, the Unified Health System (Sistema Único de Saúde, SUS), Brazil, but political disagreements between regional governments and between these and the federal government have resulted in a chaotic amalgam of NPI implemented across the country [11,12,13]. Most of these measures were gradually removed by each Brazilian state government between 2021 and 2022 using the reasoning that the worst of the pandemic had faded and most of the population was vaccinated (~67% at the beginning of 2022) [14]. Ironically, the country experienced in February 2022 its highest number of COVID-19 cases, reaching more than 298,000 cases notified in a day. Presumed causes for this rise are the disuse of nonpharmaceutical interventions (NPI) against COVID-19 at the end-of-year holidays [15], the resurgence of the well-established Delta variant [16], and the introduction of Omicron in the country in late 2021 [17,18,19].

The first case of Omicron was detected in Brazil on 30 November 2021 in the state of São Paulo, but other states quickly reported new cases. Because of the difference in the durations and austerity of NPI, it is possible that the Brazilian state limits may have functioned as barriers with different permeability to the entrance of the BA.1 and BA.2 lineages of the Omicron variant [20]. On the other hand, factors such as population density, the presence of international airports, and seasonal traveling have been demonstrated to have influenced the dispersal of lineages across the world [21,22,23] and during the first year of the pandemic in Brazil [24,25,26,27]. Understanding the spread dynamics of SARS-CoV-2 is fundamental for controlling the new variants that are expected to emerge in the future [28,29] and implementing cost-efficient, evidence-based NPI [30].

Considering this need, we have sequenced in this work 2173 genomes of SARS-CoV-2 sampled in all five geographical regions of Brazil to reconstruct the dispersal history of BA.1 and BA.2 lineages of the Omicron variant in the country. The new sequences increase considerably the number of genomes from northern, northeastern, and central–western states, commonly underrepresented in the phylodynamic analysis of SARS-CoV-2 in Brazil due to sampling bias. In addition to these sequences, we analyzed more than 18,000 different genomes deposited in the GISAID database. We demonstrate that the most populous states of the Southeast region acted as initial dispersal hubs to the rest of Brazil, although all regions have similar interstate transmission rates. We also highlight the lineage dynamics of SARS-CoV-2 in Brazil and its association with an increase in COVID-19 cases.

## 2. Materials and Methods

### 2.1. Sampling and Sequencing

Virus RNAs from nasopharyngeal or oropharyngeal SARS-CoV-2 samples from patients with positive RT-PCR diagnosis (Ct ≤ 34), collected in public and private laboratories between 30 October 2021 and 29 April 2022, were sent to Laboratório Nacional de Computação Científica for further analysis. The QIAamp (Qiagen, Hilden, Germany) or MagMAX Viral/Pathogen (ThermoFisher Waltham, MA, USA) Nucleic Acid Isolation kits and the KingFisher (ThermoFisher Waltham, MA, USA) automatic platform were utilized to extract the genetic material from the samples. Sequencing libraries were constructed at the DFA/LNCC Genomics Unit with the Illumina COVIDSeq Test (Illumina San Diego, CA, USA), according to the manufacturer’s reference guide without any modifications, starting from the “Anneal RNA” reaction. Pools of 96 libraries were created using 5 µL of each individual library and then purified. A TapeStation (Agilent, Santa Clara, CA, USA) system was used for quality control purposes and to determine the molarity of each pool of libraries. Sequencing was performed with NextSeq 500/550 Mid Output v2.5 (300 Cycles) kits in a NextSeq 500 sequencer (Illumina San Diego, CA, USA) programmed to generate 2 × 149 bp reads. The DRAGEN COVID Lineage v3.5.8 pipeline was used for sequence analysis, consensus building, and variant calling. The sequences were classified into PANGO lineages with the PangoLEARN v1.13 model database. The study was approved by the Ethics Committee (33202820.7.1001.5348), and all genome sequences were deposited in the GISAID database (doi.org/10.55876/gis8.221221hw, accessed on 21 December 2022). When samples came from minors, formal verbal consent was obtained from the parent or guardian.

### 2.2. Phylogenetic Analysis

We assembled genomic datasets representative of the pandemic in Brazil following a similar strategy to [31]. First, we assembled datasets for lineages BA.1 and BA.2 following the proportional sampling design conducted in the aforementioned work. Briefly, we retrieved from the GISAID database all BA.1 and BA.2 sequences available from Brazil and calculated the proportion of both lineages in each Brazilian state during each week between 14 November 2021 and 1 May 2022. Along with data on COVID-19 suspected cases [32], these proportions were used to estimate the number of variant-specific infections per state and week. Afterward, these estimates were normalized by the inferred total number of BA.1 and BA.2 cases, revealing the proportion of variant-specific infections for each spatiotemporal bin. These proportions were then used to assemble a genomic dataset, considering fixed target numbers of sequences (BA.1: 5000; BA.2: 1000).

To cross-verify results obtained with the aforementioned strategy, we assembled an additional dataset. In this, we employed a uniform sampling design by including a fixed number of sequences (*n* = 20) from each spatiotemporal range. When less than 20 sequences were available in GISAID, we used all of them. For all datasets, background NextStrain sequences were included, (NextStrain builds 21K and 21L, as of 18 August 2022) [33]. All datasets were aligned using MAFFT v.7 [34]. Maximum likelihood trees were then inferred with IQ-TREE v.2.0.3 [35] using the GTR+F+I+G4 model with polytomies allowed (--polytomy option). Node support was calculated with the approximate likelihood ratio test with 1000 replicates. The relationship between root-to-tip distances of the resulting trees and sampling dates was analyzed with TempEst v1.5.3 [36], and sequences that exceeded 1.5 times the interquartile range of the residuals in the root-to-tip distance distribution were removed from the dataset.

We randomly selected 10% of sequences of each lineage of the proportional dataset to infer their substitution rate with BEAST v.1.10.4 [37] using the strict clock model, the GTR model with estimated base frequencies, the proportion of invariant sites and Gamma distribution with four categories, and the exponential coalescent model. The maximum likelihood trees inferred previously and the estimated substitution rate were then used to reconstruct the divergence dates of all sequences in each dataset. For this, we used the new and faster tree likelihood algorithm available in BEASTv.1.10.5 along with the skygrid coalescent prior (60 grid points). All remaining priors and operators were used as defined in default. We ran five independent MCMC chains for the BA.1 datasets and three for the BA.2 datasets, each chain being composed of 250,000,000 steps with sampling every 25,000th. We removed a burnin of 20% of the posterior trees and summarized them with TreeAnnotator.

Finally, to investigate the dispersal rates of BA.1 and BA.2 across Brazil, we independently employed a set of 1000 random after-burnin posterior trees on a Discrete Trait Analysis (DTA) with the asymmetric rates model [38]. We coded each sequence with the Brazilian state of origin and calculated the Markov Jumps according to each state transition. The MCMC was run through 10,000,000 steps with sampling every 10,000th. We chose the median of the Markov Jumps counts as representative of the number of transitions between locations. Because transition counts will be biased towards more represented states, we also analyzed the posterior transition rates.

### 2.3. Epidemiological Analysis

We evaluated the course of the COVID-19 pandemic in Brazil between 1 October 2021, and 30 April 2022 by summarizing the number of cases and deaths caused by the disease and the number of vaccinated people, using data made publicly available by the Brazilian Ministry of Health (https://covid.saude.gov.br/ accessed on 30 August 2022). The frequency of each lineage in the analyzed period was also investigated, using both data available in GISAID and the newly sequenced genomes. Figures were generated with the *ggplot2* package in R software (version 4.1.1) [39].

## 3. Results

**Figure 1:**
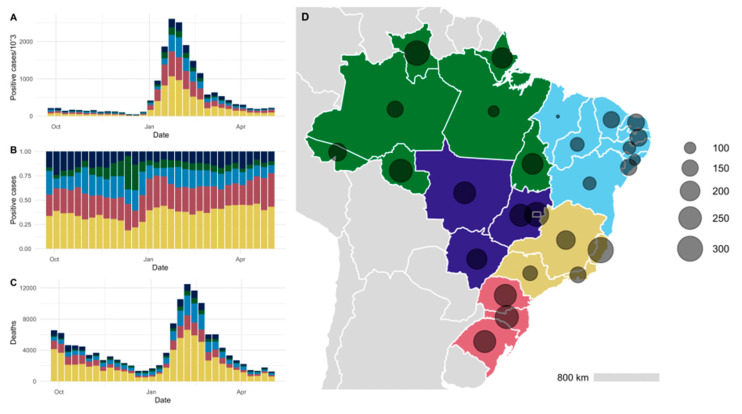
Evolution of the COVID-19 pandemic in Brazil between 26 September 2021 and 1 May 2022. In (**A**) is represented the absolute number of cases in the period, while in (**B**) this number is presented in relative frequency. In (**C**), the absolute number of deaths is shown. The circles in (**D**) indicate the number of cases per thousand people in each Brazilian state since the beginning of the pandemic until September 2022. Colors represent the regions of Brazil: North (green), Central–West (dark blue), Northeast (light blue), Southeast (yellow), and South (pink).

At the beginning of the period analyzed in this study, in October 2021, the numbers of COVID-19 cases and deaths were in clear decline, reaching the lowest numbers since the start of the pandemic in Brazil (44,566 COVID-19 cases in the third week of December 2021). By the turn of the year, these numbers started to rise again and reached a peak of more than 2.6 million cases notified in the week of 23 January 2022. This number was the highest observed in Brazil as of the submission of this work (Figure 1). The rise in deaths did not follow this sharp trend and increased to 12,492 in the second week of February, less than a third of the peak that occurred during the first semester of 2021 when the Gamma variant was spreading across Brazil. Overall, participation of each Brazilian region in the total number of cases was constant throughout the period, with a small increase in cases from the Northern region in December 2021, immediately prior to the rise in the absolute number of cases in Brazil at the beginning of 2022. Each geographical region follows the same trend in the number of positive cases and deaths.

A total of 45 Pango lineages were identified in the 2173 newly sequenced genomes. The most frequent lineages were BA.1 (709 sequences, 32.6%), BA.1.1 (638 sequences, 29.4%), BA.1.14.1 (133 sequences, 6.1%), AY.99.2 (124 sequences, 5.7%), BA.1.15 (96 sequences, 4.4%), and BA.2 (66 genomes, 3.0%). As expected, most of the lineages encountered pertained to either the Delta or Omicron Variants of Concern, with exceptions being a single P.1.7 (Gamma variant) from the state of Tocantins collected in late October 2021 and a single B.1.1.529 from the state of São Paulo collected in late December of 2021. The lineage XAG, a recombinant of BA.1 and BA.2 first identified in Brazil, was detected circulating at the end of April 2022 in the state of Rio Grande do Sul.

**Figure 2:**
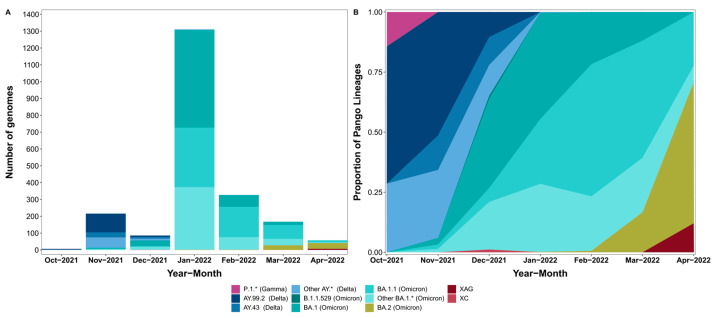
Distribution of SARS-CoV-2 lineages in this study from October 2021 to April 2022. In (**A**) is represented the absolute number of genomes by lineage and in (**B**), their relative frequency.

We highlight here that, since the first introduction of the Omicron variant in Brazil, it quickly substituted the previously dominant Delta variant. In November 2021, 94.0% of the 216 genomes sequenced belonged to Delta and only 6.0% to Omicron. By December, this proportion had already changed to 34.9% and 64%, respectively, of the 86 sequenced genomes, and by January, more than 99% of the 1311 genomes belonged to Omicron (Figure 2). In February, March, and April, only lineages associated with the Omicron variant were detected among the sequenced genomes. This replacement pattern is very similar to the one obtained by considering all the genomes from Brazil deposited in GISAID during the timeframe analyzed, indicating that our samples are representative of the pandemic in the country (Figure S1).

Using both the new genomes and sequences deposited in the GISAID database, we have reconstructed the evolutionary history of the introduction and dispersal of the lineages BA.1 and BA.2 in Brazil. We observed that multiple introductions in Brazil occurred for both lineages, with subsequent community spread in most cases. We have estimated that BA.1 emerged in the world as early as mid- to late-October 2021, and by mid-November 2021 the lineage was already circulating in the country (Figure S2A). The state of São Paulo received most of the lineage international imports (629 of 1016 import events, 95% HPD = 556–696, Table S1). The most common interstate dispersals were from São Paulo to Minas Gerais (*n* = 393, 95% HPD = 368–415) and to Rio de Janeiro (*n* = 385, 95% HPD = 361–410), followed by the dispersal from São Paulo to Rio Grande do Sul (*n* = 310, 95% HPD = 283–332).

When considering the BA.2 lineage, we observed that the lineage emerged in the world before mid-November 2022 and was already circulating in southeastern and northeastern Brazil in early January of 2022 (Figure S2B). As occurred with BA.1, most introductions of BA.2 occurred through São Paulo (136 of 277 international imports, 95% HPD = 124–158, Table S2), followed by the states of Santa Catarina (*n* = 45, 95% HPD = 41–49) and Rio de Janeiro (*n* = 31, 95% HPD = 24–38). The frequency of interstate dispersal was also similar to that found for BA.1: most common was the transition from São Paulo to Rio de Janeiro (*n* = 56, 95% HPD = 43–72), followed by São Paulo to Minas Gerais (*n* = 16, 95% HPD = 13–18), São Paulo to Rio Grande do Sul (*n* = 12, 95% HPD = 7–15), and São Paulo to Santa Catarina (*n* = 11, 95% HPD = 7–14).

**Figure 3:**
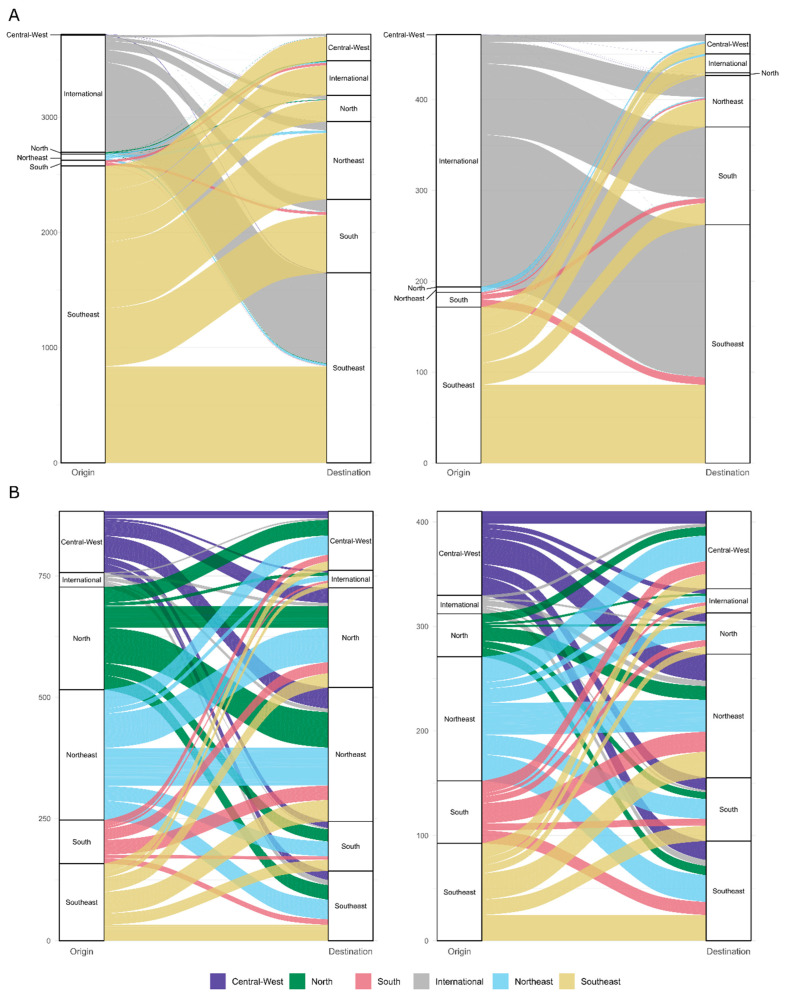
The number of introduction events (**A**) and dispersal rates (**B**) of the Omicron lineages BA.1 (**left**) and BA.2 (**right**) between Brazilian regions, estimated from the proportional dataset. Colors represent the region of the event’s origin.

Because counts of Markov Jump are influenced by the number of sequences from each location, we additionally evaluated dispersal rates between each Brazilian state. Differently to that which was observed with the count analysis, all regions of Brazil participated considerably in the interchange of SARS-CoV-2 across the country. For BA.1, the Northeast region of Brazil had the highest overall rate of interstate dispersals, closely followed by the North and Southeast regions (Figure 3). The highest values were observed in the transmission from São Paulo to the states of Minas Gerais (9.94, 95% HPD = 7.25–12.19), Rio de Janeiro (mean rate of 9.83, 95% HPD = 7.83–12.22), and Rio Grande do Sul (7.93, 95% HPD = 6.17–9.99). The overall BA.2 pattern of migration rates is again very similar to BA.1 but with a dispersion rate generally lower in the North region and higher in the Central–West region. The state with the highest import rate is São Paulo (mean rate = 3.27, 95% HPD = 2.19–4.97), and the highest interstate rates are in the dispersal between the state of São Paulo to Rio de Janeiro (9.81, 95% HPD = 6.59 = 14.35) and São Paulo to Minas Gerais (2.86, 95% HPD = 1.34, 4.64). Results with the uniform dataset corroborate the findings for both counts of dispersal events and rates of dispersal between states (Table S1 and Figure S3).

## 4. Discussion

We provide here a wide analysis of the predominance and spread of BA.1 and BA.2 lineages in Brazil, registering the moment of transition between Delta and Omicron variants to an earlier date than the first report of Omicron in Brazil, in December. In fact, the earliest Omicron sequence from Brazil deposited in GISAID was collected days prior to the discovery and announcement of the variant in Southern Africa [20]. Even though the South African research group performed outstanding work quickly identifying and noticing Omicron once it reached their borders, the early international spread of the variant highlights how genomic surveillance across the world is still failing. Specifically, it sounds an alarm about the real possibility of new variants arriving and leaving Brazil under the radar. This is a particular problem in Brazil, as genomic data from several states are scarce. For example, the state of São Paulo concentrates 40% (73,738 of 182,167) of the SARS-CoV-2 genomes sequenced in Brazil while the state of Piauí has only 0.2% (312/182,167) at the moment (GISAID, accessed on 30 September 2022). The present work has helped to bridge this gap by sequencing 592 genomes from 17 states of the Central–West, Northeast, and North regions.

During the period analyzed in this work, we observed that the substitution of the Delta variant by BA.1 and BA.2 started in December 2021 followed by a sharp increase in COVID-19 cases in January 2022. For now, the rise in the number of cases in Brazil seems to be majorly associated with the introduction and spread of new variants. It was this way with Gamma [40,41,42,43], lineages BA.1 and BA.2 of Omicron [18,44], and more recently, lineages BA.4 and BA.5 of Omicron in June 2022. As the pandemic is now expected to continue via small local outbreaks caused by a reduction of collective immunity [45,46], the cyclical growth and reduction of the SARS-CoV-2 population size is especially worrisome, as it may easily fixate new mutations and generate new variants by both selective pressure and genetic drift [47,48]. While we have not found in this work any unreported new mutations in significant frequencies, it is important to note that the Omicron sublineage BA.1.14.1 probably originated in Brazil as a reflection of the expansion of the Omicron variant at the turn of 2021 to 2022. Even more alarming is the emergence of the lineage XAG in Brazil [49], a recombinant of BA.1 and BA.2 that has spread at least to Chile, the United States of America, Denmark, Germany, and Israel. Recombinant lineages may combine the advantageous mutations of the parental lineages, posing a risk of generating highly infectious variants [28,50,51]. The coinfection event needed to generate a recombinant lineage is more common when the number of cases is high, adding another factor to the importance of controlling both the number of cases and the diversity of lineages circulating at a time.

On this last matter, we draw attention to the role played by states in the Southeast region of Brazil in the introduction of new lineages and their spread. The state of São Paulo alone was responsible for around 60% of BA.1 import events and 50% of BA.2, with the state of Rio de Janeiro accounting for an additional ~10% of imports in both lineages. São Paulo also has the highest estimated rates of dispersal to other states. While the importance of both São Paulo and Rio de Janeiro in the spatial dynamics of SARS-CoV-2 within Brazil has already been noticed in previous works [31,52,53,54,55], our results provide evidence that surveillance in both states is fundamental to the early detection of the arrival of new dangerous variants that may emerge across the world. In fact, SARS-CoV-2 testing in airports or a few days after arrival may prove to be one of the most cost-effective and important epidemic control measures in the southeastern states [56].

While the introduction of SARS-CoV-2 diversity from international sources is an important factor when considering the dynamics of the virus in Brazil, the highest dispersal rates in both BA.1 and BA.2 occurred within the country. The Southeast region acts as a hub of viral dispersal, sending the newly received haplotypes to other domestic regions. In particular, there is a trend of dispersal from the southern coastline to the northeastern one, a pattern already observed in previous research [31,52,53,54,55]. For BA.1, the North and Northeast regions act as one; they exchange viruses at a rate similar to the within-region dispersal seen in both. Overall, the observed patterns could be due to travels during the end-of-the-year holidays in 2021, as Christmas and New Year celebrations are commonly regarded as superspreading virus dispersion events. These could have caused the introduction of the Omicron variant in Brazil and fired its dispersion across other states, but further investigation is still needed to confirm this hypothesis.

It is important to note that, while this study focuses on describing the SARS-CoV-2 dispersal occurring within Brazil, one can probably extrapolate the patterns identified to the viral spread across the world. In the same way that populous cities act as hubs for the introduction and spread of new lineages, populous countries may have the same role within continents, especially if these commonly receive a large traffic of people [57,58]. On the other hand, the dispersal of variants across political borders has become significantly faster since traveling restrictions went down, which could dilute the power of a single country being responsible for starting the spread to a whole continent [58]. The first identification of BA.1 in other South American countries occurred mostly in December 2022 while BA.2 was usually first detected in February 2022 [59,60,61], both later dates than the observed in Brazil. Still, it is difficult to precise the introduction dates and the routes of spread across the continent, or even if Brazil was the source of introduction, because some regions are severely undersampled in comparison to the number of COVID-19 cases per habitant.

Finally, the results here provide strong evidence for the guided use of NPI. As the number of vaccinated people increased and the number of cases dwindled in 2021 [62,63,64], Brazilian states gradually abandoned the different measures of epidemic control employed, including the obligatory use of masks and social distancing. While it is understandable that the continued use of such interventions has impacts on public health, employment rate, and education [65,66,67,68,69], the complete removal of NPI left the country vulnerable to the entrance of Omicron, which caused the current record of COVID-19 cases in a week. Even though the number of casualties did not rise in such magnitude, an explosion of cases still causes serious economic and public health consequences due to the high number of hospitalizations and the need for treatment [70,71,72]. In particular, we would like to highlight the impact of the debilitating persistence of COVID-19 symptoms for long periods after the disease has already subsided (“long COVID”) [73,74,75] and the hospitalization risk of unvaccinated people such as children under five years of age and immunocompromised individuals [76,77,78]. Our results indicate that punctual restrictions prior to periods of intense traveling and intelligent testing at state borders and airports might mitigate the abandonment of NPI by reducing the number of import events in Brazil and each of its states.

## Data Availability

Not applicable.

## Supplementary Materials

The following supporting information can be downloaded at: https://www.mdpi.com/article/10.3390/v15040888/s1, Figure S1: Distribution of SARS-CoV-2 lineages in Brazil between October 2021 and April 2022; Figure S2: The time tree of the BA.1 and BA.2 sequences analyzed; Figure S3: The number of introduction events and dispersal rates of the Omicron lineages BA.1 and BA.2 between Brazilian regions, estimated from the uniform dataset; Table S1. Quality of sequencing and assemblage of the genomes generated for this work; Table S2: Posterior values for Markov Jump Counts and mean dispersal rates obtained for BA.1 and BA.2 in both proportional and uniform datasets.
