# Supplementary material for: The Omicron Lineages BA.1 and BA.2 (Betacoronavirus SARS-CoV-2) Have Repeatedly Entered Brazil through a Single Dispersal Hub
Source: Viruses. 2023 Mar 30;15(4):888. doi: 10.3390/v15040888 (PMC10146814; doi:10.3390/v15040888)
Supplement: Supplementary file 1 [file viruses-15-00888-s001.zip › Figure S1-S3.pdf]

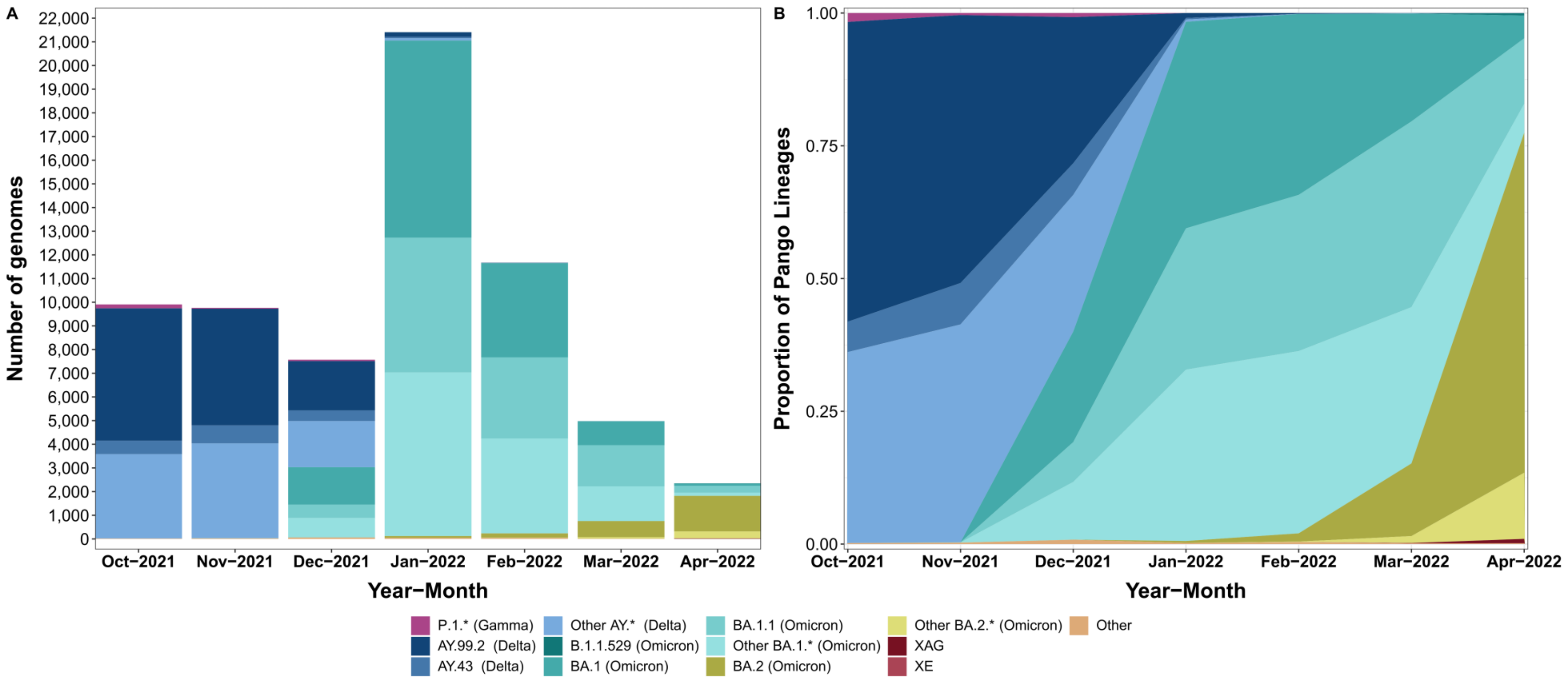

Figure S1. Distribution of SARS-CoV-2 lineages in Brazil between October 2021 and April 2022. In (A) is represented the absolute number of genomes by lineage and in (B), their relative frequency.

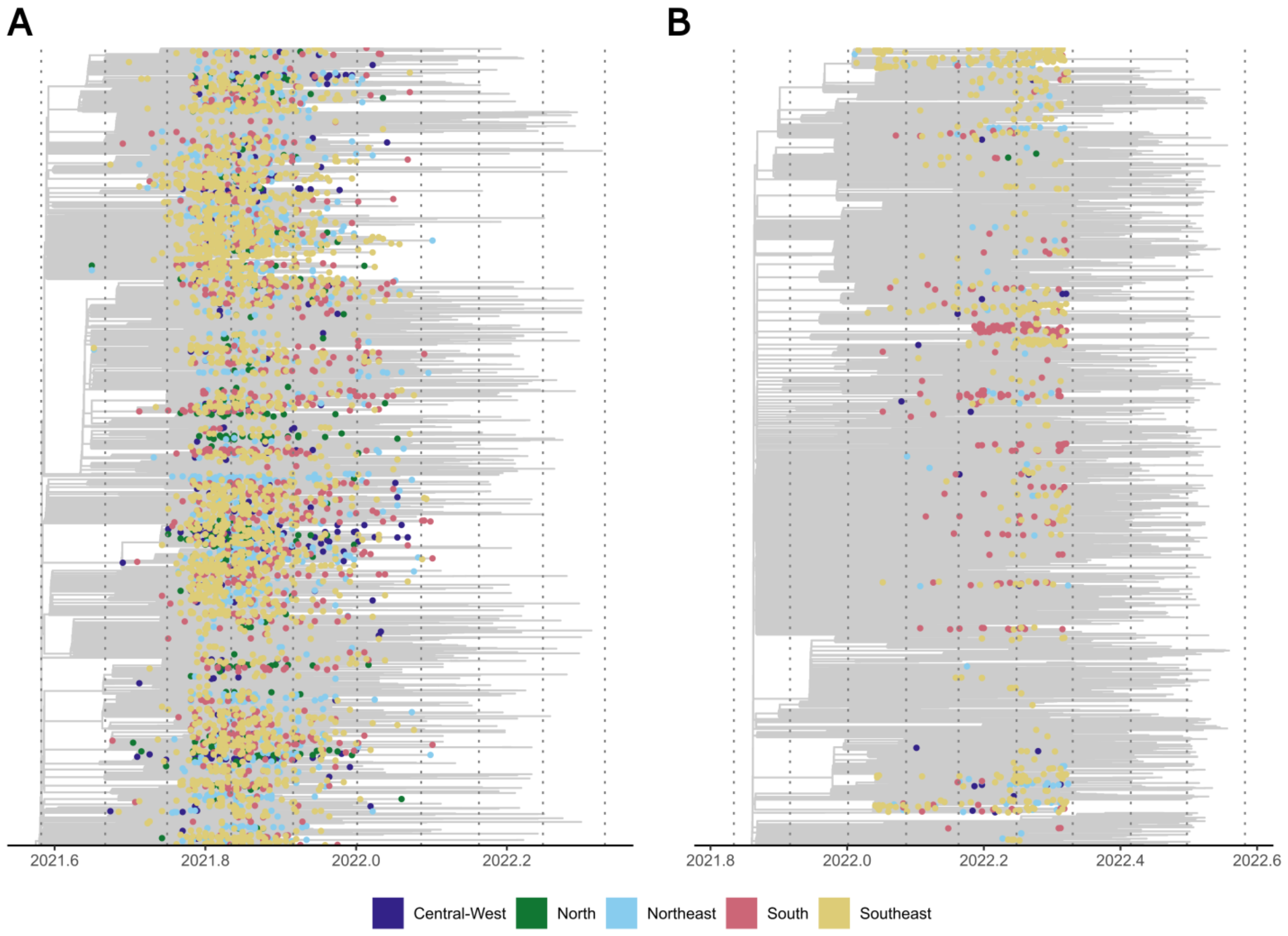

Figure S2. The time tree of the BA.1 (A) and BA.2 (B) sequences analyzed. Colors represent the region of collection of the Brazilian sequences, evidencing the multiple introductions in the country.

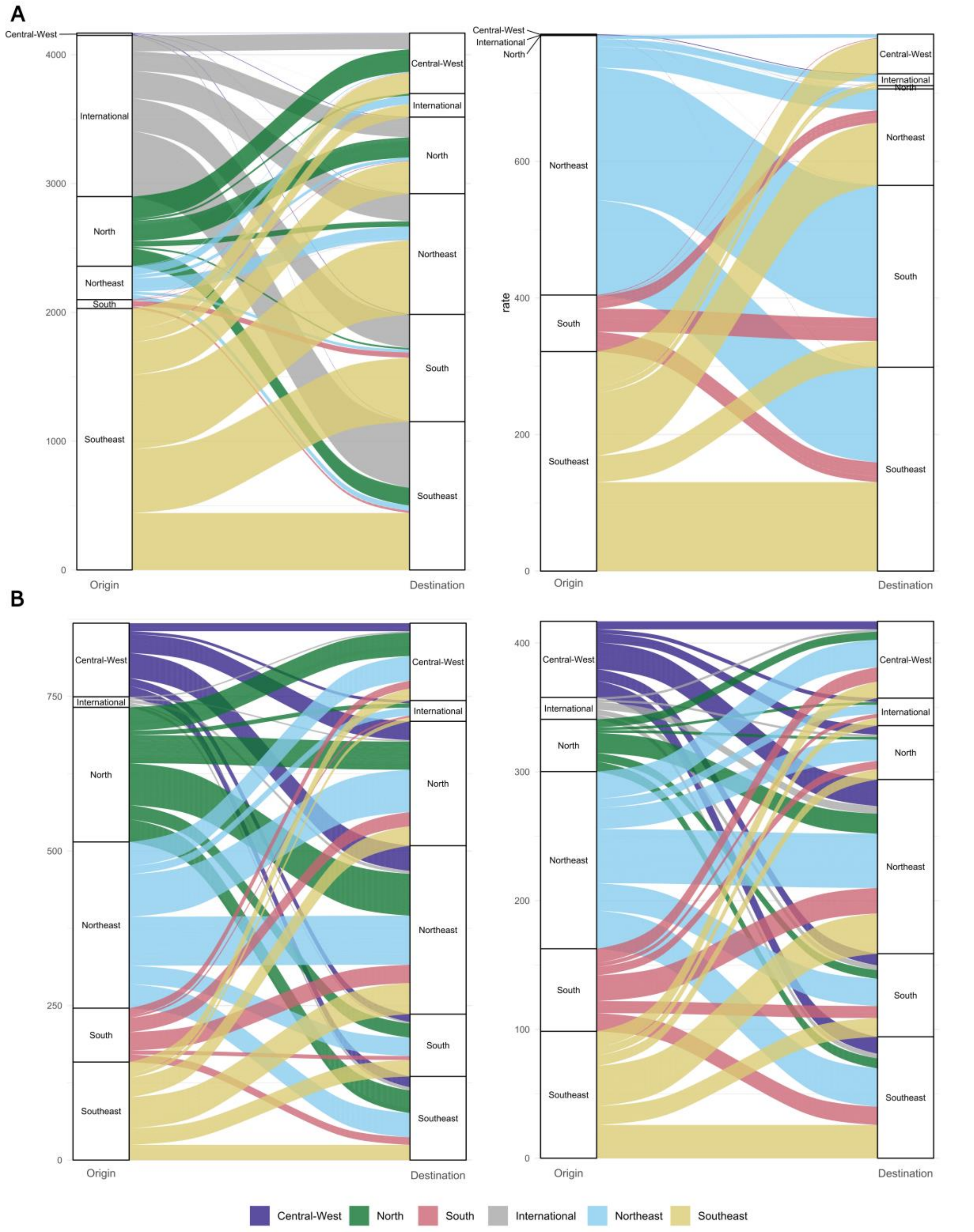

Figure S3. The number of introduction events (A) and dispersal rates (B) of the Omicron lineages BA.1 (left) and BA.2 (right) between Brazilian regions, estimated from the uniform dataset. Colors represent the region of the event's origin.
